# Supplementary material for: “What influences how well householders living in previously flooded communities feel they are protected or could recover from future flooding?: Results of a survey”
Source: PLoS One. 2024 Aug 7;19(8):e0306593. doi: 10.1371/journal.pone.0306593 (PMC11305524; doi:10.1371/journal.pone.0306593)
Supplement: Attachment — Submitted filename: Response to reviewers.docx [file pone.0306593.s004.docx]

Response to reviewers

PONE-D-23-41282R1] - [EMID:4412e0a1c626444b]

Please find below a list of our revisions as requested.

Reviewer 1 asked us to polish the manuscript. This is a rather general comment – we have reviewed the manuscript again and made some changes to sections of text for clarity.

Reviewer 1 asked that the ordinal regression results be rewritten to provide greater clarity in expression. We have used much of the material suggested by the reviewer and added this to the section. We have retained the statistical description as this was requested by a previous review. This way we hope to meet the needs of both the generalist and more statistically interested reader.

Reviewer 1 asked that we look at the conclusion and improve the writing. We have revisited this section again and removed some repetition, and hope this meets their expectations.

Reviewer 2 mentioned that the policy recommendations could benefit form increase specificity, but accepts these are acceptable. We have not revised these as given this is a secondary analysis we do not feel it appropriate to be more specific.

In addition, we have been through all the references to ensure that the links work for the electronic papers and checked that none of the papers cited have been retracted.
