# Supplementary material for: “What influences how well householders living in previously flooded communities feel they are protected or could recover from future flooding?: Results of a survey”
Source: PLoS One. 2024 Aug 7;19(8):e0306593. doi: 10.1371/journal.pone.0306593 (PMC11305524; doi:10.1371/journal.pone.0306593)
Supplement: Attachment — Submitted filename: Response to reviewer comments 22 Mar.docx [file pone.0306593.s003.docx]

**Response to reviewer comments.**

Thank you for your comments. We have sought to address these issues in our revised manuscript and feel that your comments have made for a stronger manuscript (if rather longer).

**Reviewer #1: The author should consider the following additional comments:**

1. The abstract should clearly state the objectives of the paper.

The abstract has been amended to include this aim of the paper:

The aim of this study was to examine how the impact of previous experience of flooding, demographic factors including age and gender, and behavioural actions impact on feelings of protection against flooding and the ability to recover from flooding.

2. The reader may have questions regarding the accuracy of the statement that flooding impacts more people than other environmental disasters, as well as what is meant by "environmental disasters" in this context.

We have largely rewritten the introduction and provided evidence regarding the accuracy of the statement reflecting the global impacts of flooding and used other sources in the introduction.

3. The manuscript should explore potential confounders for statistical analyses, such as religion, income, and ethnic group.

We have provided more data relating to the demographics of the sample and our local population (see results). We did collect data on ethnicity and disability but these were not significant predictors in our analyses so had excluded them to reduce the paper length. We have now included these data in the manuscript results section. We did not collect information on religion and income, to maintain a balance between burden on respondents and maximising engagement. The survey team felt justified in this approach as there were very few respondents that began the survey but did not complete it (including none online – this is recorded on the online software). We have explained this in the text including as a limitation of the study.

4. It would be beneficial to provide information on Hull's population demographic characteristics and the relationships between the population and sample demographics.

Comparisons of demographic information between the survey respondents and from Hulls population at the time of survey have been included at the start of the results section along with the demographic data from the sample.

5. Table 5 needs better explanation as it is currently difficult to understand without accompanying text.

An expanded narrative is provided in the results section as requested.

6. Consider rearranging the section titled "Strength and Limitations" to "Limitations and Strength." The aforementioned points can be discussed as limitations of the study.

We have expanded the limitations and strength sections as requested and discussed the data not collected and the demographics of our sample and local population.

**Reviewer #2: Overall, I find the study to be promising, but there are a few critical areas that require attention and improvement before it can be considered for publication. Please find detailed feedback below:**

Insufficient Literature Review and Conceptual Framework:
The manuscript lacks a comprehensive literature review, particularly in establishing a robust theoretical framework connecting demographic factors and the perceived ability to cope and recover from floods. It is crucial to provide a more detailed theoretical foundation to convincingly support the research objectives. I recommend incorporating relevant literature that directly addresses the linkage between demographic context/factors and flood resilience. This will strengthen the theoretical underpinning of your study and ensure that the research is aligned with its stated objectives.
We have largely rewritten the introduction and included relevant literature to support the hypothesis that demographic factors are associated with perceptions of being able to cope with and recover from flooding. We have drawn on evidence from a recent systematic review of UK studies which indicate a link between the experience of flooding, demographic factors and mental health outcomes in the short term (<12 months) and how more longer term evidence is needed, if we are to develop more nuanced support to communities. Our manuscript addresses this need.

Clarity in Terminology – Resilience vs. Perceived Capacity:

The manuscript often uses the term "resilience" to describe the outcomes related to the feeling of recovering and the ability to protect oneself from floods. However, resilience and perceived capacity are distinct concepts. It is advisable to refrain from categorizing the findings as contributing to "resilience" without a more nuanced exploration of these terms. Consider revising the terminology to accurately reflect the study's focus on the perceived capacity to cope and recover from floods.

We provided a discussion about the conceptual link between perceived coping and resilience to contextualise our discussion of flood resilience. We have discussed how our findings can contribute to understandings of flood vulnerability, coping, capacity and resilience (and how these link together), drawing more effectively on a range of background literature.  We think some link to resilience is important because the survey was conducted to support a flood resilience programme being conducted by the local Flood Risk Management agencies and the language aligns with current UK policy that conceptualises the communities ability to cope, (which includes perceptions of an ability) as having community flood resilience.

Illustration of Hazards and Challenges for Different Demographic Groups:
The extent of hazards and challenges faced by different age groups and genders is not sufficiently illustrated in the current manuscript. To enhance the understanding of the difficulties experienced by various demographic groups, I recommend providing a more detailed analysis of the hazards encountered and their impact on different age groups and genders. This will contribute to a more nuanced and comprehensive interpretation of the study findings.
Related to our responses above, we have tried to achieve this by expanding the background literature to include evidence on the impact on different groups (focusing on age and gender in detail) and introducing more material into the discussion to consider who different demographic groups face different challenges.

Enhanced Policy Recommendations:

The policy recommendations lack a direct and explicit link to the discussion. Instead of suggesting "specific work" or "practical action," consider providing concrete and targeted policy recommendations that align with the research findings. For instance, during the flood prevention phase, consider suggesting measures such as increasing the experience of female and elderly populations through flood drills or simulation exercises. Additionally, consider conducting short interviews to identify specific concerns among these demographics, allowing for more precise and actionable policy recommendations. Such detailed and context-specific suggestions will enhance the practical utility of your research, particularly for the city of Hull.

Thank you. We have expanded on our policy recommendations, including making a more direct link to the collaborative work in Hull. We have also strengthened the link between the discussion section and conclusions. We also found a specific reference critical of the UK government lack of focus on gender disaggregation in disaster policypost and have woven this into the paper. The reviewer comments around practical activities are very helpful and we have incorporated these into our paper.

I appreciate the effort you have put into this research, and I believe that addressing these concerns will significantly strengthen the manuscript. I look forward to reviewing a revised version that incorporates these suggestions.

Thank you for your supportive comments.
